# How Shall We Manage Abortion?

**Published:** 1886-01

**Authors:** M. Hartwig


The Buffalo Medical and Surgical Journal

Vol. XXVI. JANUARY, 1886. No. 6.

### Original Communications.

### How Shall We Manage Abortion?

By Dr. M. Hartwig.

Read before the Buffalo Obstetrical Society, November 24, 1885.

In considering my subject, let me follow the two latest essays of E. Schwarz, Halle Volkmann's Sammlung Klinischer Vortræge, No. 241, abridged in *Central-Blatt fuer Gynæcologie*, No. 1, 1885, and Kleinwæchter article, "Abortus in Eulenberg's Real Encyclopædie der gesammten Heilkunde," and divide the subject into Prophylaxis and Therapy.

Kleinwæchter says in his article, which is a model of its kind: "The prophylaxis of a healthy woman is to avoid all noxæ which are liable to interrupt the pregnancy, and cites in a most exhaustive way all of them, but this is not our object. He further says: "With a sick woman, caution must be redoubled. If there be a feverish condition present, the temperature should be reduced, but we seldom succeed." From my own experience, I can cite only two successful cases which I have had of late. The first summons I had was on account of violent pain in the abdomen of a multipara in the sixth month. Evidently, labor pains, high fever, and astonishingly lively movements of the fœtus were present. I made a subcutaneous injection of grain 1/8 of morphine, and ordered antipyrin in sufficient doses to reduce the fever. The condition of the patient, almost resembling a peritonitis, lapsed quickly into a distinct condition of typhoid, with roseola and swelling of the spleen; while the labor pains vanished entirely with the same therapeutic measures, and after three weeks the woman was well, the pregnancy continued up to date. Quite an interesting question is suggested, "Had this fœtus typhoid fever, and is it sure never to get it in future life?" Another case I found in my records—typhlitis with twin pregnancy—where I succeeded by proper treatment to see the confinement at term, after the typhlitis had lasted eight days. The other day I saw a case of the reverse, apparent puerperal fever third day after premature confinement, while it actually was a pneumonia which caused the premature birth. Kleinwæchter further says: "Constitutional diseases we have to combat, especially syphilis, where we can achieve the best results. When dislocations of the uterus, or other diseases of this organ, are present, they must be treated with especial care." In this respect, I can cite from my experience a few cases. One was a retroflexio uteri gravidi in the fourth month. Ischuria paradoxa was present for six weeks; pains were already very lively. With the patient in knee-elbow position, I succeeded, after emptying an enormously filled bladder with the catheter, in replacing the uterus by pushing in the laquear posterius. The woman was fed in bed for six weeks, and with the use of morphine soon recovered and continued pregnant. Another similar case in the third month was successful in the replacing, but I am doubtful whether the pregnancy continued. In one case of retroflexio, I had replaced the uterus, and the woman wore a ring. Gravidity ensued while she was wearing the ring. After the fifth month, I removed the ring, and the woman reached full term. Schwarz *l. c.* advises against reposition when the uterus is fixed by adhesions, but I doubt whether they will be often recognizable, because the uterus is at this period so large and heavy as to appear immovable. Larger operations during pregnancy ought to be avoided. They are liable to cause abortion (*vide* Matthew D. Mann, Transactions of the American Gynæcological Society, VII., 1883). I have performed only one operation during the third month, an ovariotomy. The woman continued pregnant for two months; she lost a member of her family, slipped on the street, falling heavily and bruising herself considerably, and miscarried. Teeth I have pulled fearlessly and without harm, but in very nervous women I should advise narcosis to be employed. Of other diseases of the uterus in pregnancy, I have only one of extensive erosion of the os with laceration, a condition which Mundé and Grandin cite as predisposing to abortion. I treated the patient by applying the nitrate of silver stick to the os, and she miscarried. I cannot help thinking that the silver caused the abortion, because the woman had afterwards a child at full term without having undergone further treatment. Not in every case does such a result follow the application of nitrate of silver, because in one case, at least, I applied a ten per cent. solution to the os for ten minutes on two successive days, on account of intolerable vomiting, and no miscarriage followed. This is, according to Karl Braun, of Vienna, the best means to check the excessive vomiting in pregnancy.

Kleinwæchter continues, "When the so-called abortive tendency exists, Simpson recommends chlorate of potash, grains xv., three times a day, and Jenks the fluid extract of viburnum prunifolium, half to one teaspoonful three times a day," while he himself considers these cases as being mostly syphilitics. I myself have seen one case only—a woman who had miscarried five or six times, every time about the beginning of the seventh month. During the last pregnancy, her physician had kept her for several months in bed. It did not help matters. I myself had to witness one more of her miscarriages. I suspected syphilis, used every reasonable means to ascertain its existence in husband or wife, who were very desirous to get a living child, but to no effect. In the face of absolute denials and of utter lack of objective signs, I did not dare to institute an antisyphilitic treatment of the mother, and now I regret it. In such cases, the examination of one fœtus by a good expert should be insisted upon. As far as I am able to remember, the fœtus of this couple had syphilis, and the yellow line of Wegner on the epiphysis was present, but here my memory fails somewhat, and I have no notes of the case. Thus it proves little.

Now to the abortion, which not only threatens, but which has already started. Schwarz *l. c.* expects the abortion to go to a finish with certainty whenever the cervix of a primipara is fully dilated, the ovum resting upon the external os; whenever in a multipara, the finger can pass the os internum; whenever pieces of decidua have escaped, and the discharge is of a putrid character, or when a decided bleeding has persisted for several days, or when death of the fœtus can be ascertained. To this I make one objection: The bleeding may have been considerable for several days, and occasionally we succeed in checking abortion anyway. I recollect two cases in my own practice, both in the fifth month of pregnancy; the bleeding in the second case, especially, was quite considerable, and extended with interruptions over a couple of weeks. The means employed were, essentially, rest in bed, and fluid extract of ergot fifteen drops, three times a day, with small doses of morphine. In due time, I confined the woman, a healthy girl being born. The placenta had a rusty spot on its edge, a remnant of old extravasation, showing where the edge was detached five months ago. Kleinwæchter seems not to entirely agree with me. He says, "As soon as premonitory symptoms appear, put the pregnant woman to bed. Internal medication is superfluous. Frequent short cold irrigations of the vagina may check the bleeding. The pains can disappear, and the os which was opened can close again. When success seems assured, the woman ought to be kept in bed a few days longer." To this statement I would take exception in regard to avoiding medication. It may be that it was often unnecessary, but I should rely on theoretical grounds and practical experience—as small as I have had—on small doses of morphine (*vide* Isham, Treatment of threatened abortion by hypodermic injections of morphine: *American Journal of Medical Sciences*, January, 1873, page 109; three successful cases); although I would not object to the addition of fluid extract of viburnum prunifolium. Has any one of you tried the boasted aletris cordial?

As to treatment, as soon as the conviction is reached that the progress of the abortion cannot be checked, I coincide in the main with Kleinwæchter, and thus let us follow him again. He says: "If the loss of blood be small and the general condition of the woman good, we should abstain from activity and satisfy ourselves with disinfecting injections in the vagina. If the ovum be already within the collum uteri and the os to some extent dilated, we can sometimes finish the expulsive attempt of nature by compressing the uterus bimanually (Hœnig's method), but we should abstain from all vigorous measures, or else we might rupture the membranes, see the amniotic fluid and fœtus escape, and then we would have to deal with much more difficult symptoms." As often as I myself have attempted Hœnig's proceeding, I have succeeded only once, in the Old Country, and the effect was very gratifying—the immediate end of all trouble. It seems that all connections between the secundines and the uterus must be pretty well loosened if the process shall be successful. Kleinwæchter is of the same opinion. He adds, and here I would take exception, that subcutaneous injections of ergotin, one to five of water, may be serviceable when nothing else can be done. I do not think the woman should be plagued with the additional pain of these injections; if ergot is to be used, give it internally. T. Spæth, Compendium der Geburts-Kunde, page 299, with his large experience, denies having ever seen ergot promote abortion. I recollect a woman who had taken, four times a day for four weeks, one teaspoonful of the fluid extract to induce abortion and she failed completely. Ergot is liable to contract the os internum, and could stop the progress of abortion. For this purpose I have already recommended it in doses of fifteen drops of the fluid extract three times a day, and cited a successful case. Here (I speak of cases where the idea of possibly checking the abortion has been abandoned) the ergot has, to my mind, no more use, except *after* extrusion of the ovum, as soon as we are convinced that the uterus is empty.

"As soon as the bleeding becomes profuse, expectant treatment should be superseded by vigorous measures," says Kleinwæchter. Schwarz coincides, and I take the same position. The weight of authority everywhere is with us, although an abortion before the twenty-eighth week has hardly ever caused bleeding enough to cause death ; but chronic anæmia, with very lasting weakness, is frequent. As to the means employed, only differences of opinion exist. The main distinction is to be drawn according to the question whether the ovum is whole yet or already bursted, and whether any decomposition of the uterine contents does or does not exist; and in case decomposition, *id est* fœtid discharge, is present, whether the mother's organism participates with fever, inflammation, etc., or not. Evidently, Kleinwæchter thinks of the first condition, ovum whole, no decomposition present, when he recommends the tampon to check the bleeding; and I agree with him fully. He rejects the colpeurynter; I never used it. He recommends tamponing with absorbent cotton, dipped in five per cent. carbolized oil, carefully applied to the os, and without distending the vagina too much. I should prefer carbolized glycerine of the same strength, because oil does not disinfect safely. Let me add here that I always deplore the lack of *definite strength* of antiseptic cottons in the American market. A five per cent. salicyl or iodoform and, perhaps, borated cotton, might be sufficient; the last is better when of ten per cent. strength. Frequently we find, after about six hours, the whole ovum lying behind the tampon. Now we should follow with syringing the vagina with a two per cent. or stronger solution, is Kleinwæchter's recommendation, and with the same if expulsion has not taken place. Repeated tamponing Kleinwæchter objects to. Schwarz recommends three repetitions of injections of antiseptics (105 degrees hot, or very cold) in the meantime *into* the uterus. I would not dare to follow Schwarz neither with the repetitions (unless circumstances would not admit of anything else) nor with the intra-uterine injections, because these have caused death repeatedly when artificial abortion was attempted in this way. This method of Cohen's is fallen in deserved disrepute, and should not be brought into practice again for accidental abortions. Whether Kleinwæchter's way of injecting disinfecting injections into the vagina merely after tamponing is sufficient security against puerperal fever, is doubtful to my mind. I should not neglect to cleanse the uterus itself. Here the syringing is harmless, because the os is open. Should it be contracted already, every precaution should be taken to insure the return flow of the fluid.

Once I had to pay dearly enough for such neglect, having been unable to wash the uterus in time. Having just found the whole ovum behind the tampon, I cut it open, finding the amniotic fluid and one inch of cord while the fœtus was absorbed. Now, I was going to proceed with washing the uterus, when a very urgent call led me away. Reminding myself of the antiseptic character of my tampon and of the clean character of the whole proceeding on my part, as well as of the non-putrid appearance of the ovum, I hoped for an uninterrupted recovery, notwithstanding my neglect of my usual precaution to cleanse the uterine cavity; but, alas! on the third day, slight chilliness and a measure of fever followed, while the parametrium became sensitive and somewhat swollen. I had to deal simply with parametritis on one side. Quinine and intra-uterine washing, with four per cent. carbol., cured the case, but fourteen days passed by before the woman was well. The parametritis finished with absorption, and the cure was so perfect that I confined the patient later on at term.

The second condition we meet, and one we probably meet most frequently in practice, is after the fœtus has escaped while the secundines are partly or wholly retained, and bleeding continues profusely, signs of putrefaction or sepsis being absent. In former times, expectant treatment was the plan pursued in these cases, though Osiander already thought of more active proceedings. Only since the sponge-tents came into use has the emptying of the uterus purposely been undertaken, Martin first, James Y. Simpson second. The results were not gratifying enough, because finger and placenta forceps are two clumsy instruments, while the sponge-tents themselves often induced sepsis.* A new era seems to have been inaugurated by Boeters' *Centralblatt fuer Gynæcologie*, 1877, page 352, who published ten successful cases of scraping out the secundines with Simon's sharp spoon, and consecutive cauterising and disinfecting the uterine cavity without dilating the os with tents. Boeters clearly defined this proceeding as a means to prevent putrid decomposition of the secundines. He was quickly followed by a host of followers and few opponents. To-day his proceeding, so short a time since its publication, can be deemed safely founded, and publications of the successful use of the same are already very numerous.

About the inefficiency of the former method of handling the contents of the uterus, I can cite quite an interesting example. A woman who was wearing an intra-uterine stem for anteflexio, and, perhaps, to prevent pregnancy, became pregnant all the same, (only a few cases of this kind are on record,) and abortion followed. A two months' fœtus escaped; the secundines were retained. Next morning, patient had temperature of 106° F.; I called Dr. Mynter in consultation, who tried to empty the uterus with finger and forceps, the mouth of which seemed patulous enough. Fearing he had not fully succeeded, he called the late

* With those, the same fault prevails as mentioned with the cotton.

Dr. James P. White, who repeated the proceeding. We followed with injections of four per cent. carbolic acid, and for twenty-four hours I kept a drainage tube in the uterus, a proceeding of which I am the originator, though Schede published it first. On the fourth day, two fœtid pieces of placenta came away, and convalescence began. After eleven days, the fever was over, but a salpingitis purulenta seemed to have remained, from which, three months later, a peritonitis in the left hypogastric region started, which in its turn in all probability caused metastatic putrid pneumonia. After two months, there was final recovery. A soreness over the left iliac region was pressed, and had the patient not died later of carcinoma, the inflammatory remnants could have caused mischief again.

This is a good sample, showing the enormous responsibility we carry in attending abortions, because, with proper treatment, we can prevent a score of diseases, as well as death. One of the most important means to this effect is the antiseptic scraping of the uterine cavity introduced by Boeters. It seems to do all that scientific treatment could possibly furnish; it looks like the acme of obstetric wisdom, drainage following or not. The proofs in behalf of this proceeding are already overwhelming, and it might seem unnecessary to add my own experience, but the experience of the reader is more convincing to the hearer than that of others much more extensive or numerous. I shall give, therefore, my own, but I will first consider the third condition, the presence of septic or inflammatory complications at the time your help is called for, or developing during your attendance, while the secundines are yet in utero.

For several years I had my doubts whether active interference was called for under these circumstances; but I am getting over these doubts. Of course, the conditions met with, where the guidance of the abortion for a time was not in your hands, are very variable, and sound judgment will be necessary, simply common sense, but certain cardinal rules will be always admissible, and these are: As long as there is the tithe of a show that, with thorough removal of the secundines and antiseptic cleansing, the cause of the septic, that is to say, inflammatory condition, can be *wholly* removed, the scraping ought to be proceeded with. In doubtful cases, give the patient the benefit of the doubt, and only when death is imminent, where the patient could not possibly bear the narcosis, or where there is certainty of phlebitis of the vena cava, abstain. I might have possibly saved two lives had I had courage enough.

I was called to see a woman with fully developed general peritonitis, where, three days before, the fœtus had escaped. Probably this was a criminal case. I abstained from emptying the uterus, and three days later the woman was a corpse. Now it may be thought that the woman was doomed by all means. But why did I succeed in saving a pastor's wife, who was confined by a midwife, and whom I found in the same condition, peritonitis hardly less grave, by cleansing the uterus with antiseptics? It seems to me that, before the peritonitis is well established, the process of cell activity, on the part of the organism, is able to destroy the (organized) poison, and a cure is then possible, when a fresh supply from within the uterus is interrupted; or else no wound fever could finish with a cure.

The other case I might have saved, perhaps, had I been more daring, was a woman I saw in consultation with Dr. Hauenstein. Puerperal fever of 105 to 106°, without local tenderness, without fœtid discharge — with hardly any discharge, in fact. Confinement by midwife five days before my arrival, three days before Dr. Hauenstein's. Inasmuch as there was no local tenderness, the uterus well diminished in size, and the whole process, so to say, running in the blood, while discharge was insignificant, I thought of the possibility that we had to deal with an unusual form of puerperal fever, not depending upon putrefactive germs, but of those of erysipelas, scarlet or of some other kind of puerperal fever proper, which Fordyce Barker seems to assume, and Engelmann, too. Thus I accepted Dr. Hauenstein's view, that cleansing *this* uterus would be useless, and we let the woman die without attempt to disinfect the uterus. To-day I should not follow this plan. I had no post-mortem; if we had, it might have shown some decomposition in the uterus. If cleaning the uterus should not help in such a case, it is certainly harmless. After abortion, the essentials would be just the same, only the removal of the remnants is most easily and thoroughly accomplished with the curette (scoop); that is all the difference there is. But the scoop has its value after a timely birth, as I will show shortly. Now I wish to emphasize only the thorough disinfection, and show the possibility that this case may perhaps have been saved. I had an analogous case. A woman whom I confined myself with forceps without extraordinary difficulty, where placenta and membranes entire followed, — a living child, — started on the third day with high fever, without local inflammatory symptoms, without putrescence of the lochia. Vigorous repeated antiseptic washing and drain cured her in a few days. I think I must have carried the poison some way to the patient myself, notwithstanding my usual scrupulous cleanliness in confinements. Another woman whom I confined a few days previously, had become sick, too, though the birth was natural. My belief in my own cleanliness, conjoined with the fact that I had examined her only twice, and that without manipulating extensively or long, prevented me from using local antiseptic measures, and the poor woman had to suffer for it, going through a tedious process of parametritis on both sides, followed, after an interval, by psoitis, which I opened, finishing the chapter of suffering. Where the intense and everlasting poison came from, remained a riddle to me, but you see the difference which vigorous antiseptic cleansing produces: the patient who was last attacked was well a long time, while the first had continued to suffer. Altogether, my experience with antiseptic cleansing of the uterus, after timely confinements with sepsis or puerperal fever, is highly gratifying. Why should the same not occur in premature deliveries? All the difference there is after untimely confinements in the first few months, is that only the scoop insures perfect cleansing of the uterus; though you may succeed even without it, as I could in a case of abortion in the second month, where I had reason to suppose that the secundines were discharged wholly when I was called. A few injections of four per cent. carbolic acid had sufficed to cure the fever, which had started without local tenderness. Of course, as heretofore mentioned, not everywhere the scoop and antiseptic cleansing will suffice. The poison may have pervaded organs in the neighborhood of the uterus, the lymphatics or veins of the ligaments; and, further up to the cava, embolisms of the lung, etc, may be present at the time you are called; but antiseptic cleaning cannot do harm if it is properly executed, and not only cuts off the fresh supply of poison to the system, but it seems even that the amount of medicine introduced in the uterus is partly absorbed, following the poison in its adjoining channels, where it combats it yet to a certain extent; but the main fight against the poison is here accomplished by the cells of the living organism and the process of oxidation. Too plentiful absorption of medicine has caused medicinal poisoning in many cases, and only one medicine is innocuous — one-third to one-half per cent. salicylic acid solutions.

Even after timely confinements, the scoop has found its advocates. Sternefeld and Pick, *Archiv fuer Gynæcologie*, Bd. II., Heft 2, and *Deutsche Medicinische Wochenschrift*, 1883, No. 50, and Plœnnies, in *Centralblatt fuer Gynæcologie*, 1885, No. 2, are lively advocates of the scoop, post-partum *maturum*, in cases of bleeding retention of placentapieces or membranes, and puerperal endometritis. Sternefeld lost three out of five cases of puerperal septicæmia; operations as late as the tenth day; Plœnnies, none in five cases, and Pick, one.

The details of curettement after timely confinement will have to be determined elaborately, but we have a good showing already. What I have said so far, will show that there is no essential difference left for the practical handling, whether we have to deal with bleeding after escape of the fœtus, with retention of secundines, or with decomposition of the latter as well. Scooping and antiseptic cleansing will become the rule of the future, and the expectant plan will be probably reserved for those cases only where the ovum did not burst, or where bleeding is insignificant, sepsis and fever absent. Truly, a case might run to recovery after long retention of the secundines, and without puerperal fever supervening, but we cannot rely upon this, whereas the antiseptic, actually perfect cleansing gives positive security. I have twice seen a retention of placenta for three months with copious bleeding coming to a favorable issue spontaneously, though weakness and anæmia lasted very long in one; the literature abounds in such cases. But what is this to security? How positive this security, I shall presently show from literature, as well as from my own experience.

Alloway, *American Journal of Obstetrics*, Feb., 1883, cites five successful cases; Mundé, in the same number, thirty-one cases with one death, septicæmia being present at the time of curettement. This showing is the more remarkable as six cases were feverish at the time of curettement. Farr, in the same journal, Sept., 1883, cites sixteen cases, all successful; one, operated during inflammatory process, passed through tedious peritonitis. Samuel L. Jepson, Wheeling, W. Va., reports two cases of long retention of placenta, one hundred and fifteen and sixty-six days respectively, where, finally, tupelo dilatation and curettement led to successful issue.— *American Journal of Obstetrics*, Oct., 1883. Why not operate at once, remarks the editor Mundé, if, after so long time, operation becomes necessary anyway, while, meantime, the general health suffers considerably?

S. Henningway, New York, same journal, April, 1884, page 363, reports thirteen cases without untoward result; Mitchell, same journal, June, 1884, reports eight cases where success was complete by the use of Loomis' forceps, which acts to a great extent like a curette, each spoon of the same being introduced separately and rotated around the placenta, detaching the same from the uterine wall. Not until then the blades are closed, and the ovum resp. is the placenta extracted. Somewhat similar use of a forceps, mainly as a dilator, Dr. Swayne recommended in the *Lancet*, Aug. 19, 1871. Thus one journal alone, within in a few years, gives us an array of seventy-five cases with one death only, not caused by the operation. Instead of troubling you with further literature, I shall abstain, because my essay is growing too long. The artificial abortion belonging to the scope of the same I shall drop likewise and reserve it for some future time. Only the execution of the curettement, which might interest many, I cannot leap over, though, in the main, I refer to Mundê *l. c.* I, myself, have used narcosis every time, worked with Sims' speculum, while holding the os with his sharp hook. In each case, I could introduce the curette without previous dilatation of the os. I presume this will be so everywhere, because with a perfectly closed and rigid os, the indication for curettement will be almost always wanting. Should this rare exception occur, I would prefer the extemporaneous dilatation with metallic dilators. It is desirable to possess several sizes and forms of curettes; they should be sharp to a certain extent. Perfectly blunt curettes may be insufficient in cases of very intimate adhesions. It might be useful sometimes to have one with a flexible stem, as Dr. Julius Haffmann described in the German medical paper which I was issuing, to enter flexed uteri, but generally the pulling on the portio with the hook, or Muzeux's forceps, will sufficiently straighten the channel. The corners of the uterus should be well cared for, and generally the spoon should be revolved once or twice around the wall of the uterus before withdrawal. Sometimes entering once, brings then the whole contents of the uterus. In case of doubt, go in again and again, until every crevice seems to have been scraped, and the spoon brings only a little blood. I do not believe the proceeding would ever be safe without subsequent antiseptic cleansing. My own consisted in all cases in injecting Routh's solution, iodi, ʒ; kalii iod., ʒ ii.; alcohol, ℥ ii.; aquæ, ℥ ii.; with due insurance for the immediate return of the fluid. Then I washed the vagina with 2½ per cent. acid carbol. solution. Swabbing of the uterus with cotton, or sponge, dipped in this or any other medicine, I deem insufficient, because with a narrow os the fluid is squeezed from the sponge before you reach the fundus. After the fourth month, I should fear possible absorption of too much iodine, and would use half per cent. solution of salicylic acid, which never failed at full term to insure asepsis in all cases where I had to remove the placenta with my hand, while it is fully harmless. Such a solution without alcohol is imperfect, but the flakes, swimming in the water, insure the result by attaching themselves to the uterine wall and exerting a protracted action.

My cases were as follows:

1. Seen in consultation with Dr. Packwood. The woman had bled long and profusely; fœtus escaped. Result perfect. Hysteria remained as before. No fever followed. Timely confinement since.

2 and 3. Woman who had induced abortion herself twice; copious hæmorrhage; curettement each time easy. No bad sequelæ. The second pregnancy followed the first in less than half a year, and shows that the proceeding is harmless in respect to subsequent conception, though I used the *sharp* spoon. Perfect recovery without untoward symptoms.

4. A case in consultation with Dr. Packwood; had lost a great deal of blood; perfect uninterrupted recovery.

5. As abortion began, bleeding was limited in amount, and I did not examine. For eight days I waited, with examination. Now the bleeding began to be a little too copious, but the main thing was, the discharges were becoming fœtid. *Now* I examined, and found the ovum partly protruding from the os. Hœnig's method (*à la Credé*) did not work; curettement with Dr. Bode. It took a full hour and numerous introductions of the spoon before I gained the conviction that the uterus was positively empty. The intimacy of connection of placenta and uterus was perfectly astonishing. Result, uninterrupted recovery. The most interesting fact in this case of great scientific value was, that that part of the secundines (the fœtus was gone) which protruded into the vagina (bear in mind she was never examined, and the abortion, I am convinced, spontaneous), was putrid, the intra-uterine not. This fact shows that a uterus which was not tampered with, does not contain germs of putrefaction,* while the vagina does; at least it is so in pregnancy. Most cases of missed abortion show no putrefaction, and corroborate this fact.

These samples could be multiplied, but it is not necessary. Of course, we can hardly ever exclude the possibility of previous meddling with the contents of the uterus. Decomposition of the placenta does not prove anything to the contrary.

* *Vide* "Garrigue's Missed Abortion, *American Journal of Obstetrics*, Sept., 1884, where the whole ovum was retained five months, but, finally, after repeated bleeding, expulsion. Placenta retained 123 days. Repeated bleeding; spontaneous expulsion."——*Medical Times and Gazette*, April 18, 1868:—"Retension of ovum five and a half months; tormenting hemorrhages. No putrescence; some irritative fever."——*Dublin Quarterly Journal of Medical Science*, August, 1870:—"One case is known where a lady traveled from the East Indies to England with a retained placenta; post abortum."

